# Draft Genome Sequence of *Pseudomonas hussainii* Strain MB3, a Denitrifying Aerobic Bacterium Isolated from the Rhizospheric Region of Mangrove Trees in the Andaman Islands, India

**DOI:** 10.1128/genomeA.01527-16

**Published:** 2017-02-02

**Authors:** Shubham K. Jaiswal, Rituja Saxena, Parul Mittal, Ankit Gupta, Vineet K. Sharma

**Affiliations:** Metagenomics and Systems Biology Laboratory, Department of Biological Sciences, Indian Institute of Science Education and Research, Bhopal, Madhya Pradesh, India

## Abstract

The genome sequence of *Pseudomonas hussainii* MB3, isolated from the rhizospheric region of mangroves in the Andaman Islands, is comprised of 3,644,788 bp and 3,159 protein coding genes. Draft genome analysis indicates that MB3 is an aerobic bacterium capable of performing assimilatory sulfate reduction, dissimilatory nitrate reduction, and denitrification.

## GENOME ANNOUNCEMENT

India is a vast geographical region with varied habitats and ecological niches, harboring a myriad of microorganisms with unique and useful genome characteristics (1, 2). The Andaman Islands in India nurture a huge population of mangroves, consisting of almost 50% of global mangrove species known to date (3). Bacterial species mainly from proteobacteria and actinobacteria have been isolated from the rhizospheric region of mangrove trees in India (4, 5). Their enhanced tolerance to high salt and pH are unique features which provide the impetus for their in-depth genomic studies. *Pseudomonas hussainii*, a Gram-negative rod-shaped proteobacterium, was first reported to be isolated from the droppings of a seashore bird in Taiwan, however, its genome characteristics are poorly known (6).

*Pseudomonas hussainii* strain MB3, reported in this study, was isolated from a water sample collected along the rhizospheric region of mangrove trees in the Andaman Islands in India (11.50°N, 92.70°E). MB3 was isolated on a Difco Nutrient Agar medium (Becton, Dickinson and Company, USA) at a temperature of 37°C under aerobic conditions. Colonies appeared within 24 to 48 h of incubation and were large, irregular in shape, transparent, and light brown in color with a rough texture. Pure culture was used to isolate the genomic DNA through the phenol-chloroform extraction method.

The 16S rRNA sequence of MB3 showed 99% sequence identity with the 16S rRNA sequence of *P. hussainii* strain CC-AMHZ5. Genome sequencing of *P. hussainii* was performed using an Illumina NextSeq 500 sequencer (Illumina, San Diego, CA, USA) by employing 150 bp paired-end sequencing. The genome assembly was performed using 1-Gb high-quality sequence data (Fastq format) comprised of 3,075,160 reads by employing SPAdes v3.9.0 (7). The complete draft genome is 3,644,788 bp and comprised of 41 contigs. The *N*_50_ and average length of obtained contigs were 279,963 bp and 88,897 bp, respectively. The MB3 genome has a G+C content of 59% (8) and consists of 3,159 protein coding genes, three rRNA genes (5S, 16S, and 23S), and 53 tRNAs identified using Glimmer v3.02, RNAmmer v1.2, and tRNAscan-SE v1.3.1, respectively (9–11). The functional annotation of genes was carried out using BLAST v2.2.26 against the NCBI nr database (12). The metabolic pathways were identified using the KEGG automated annotation server (KAAS) (13). The genome contained an entire set of genes for glycolysis, TCA cycle, and oxidative phosphorylation and hence, was concluded to have aerobic metabolism. The presence of *CysND, CysC*, *CysH*, and *CysJI* genes grants an assimilatory sulfate reduction function to the MB3 genome. Furthermore, MB3 is capable of carrying out dissimilatory nitrate reduction using genes *NarGHU*, *NapAB*, and *NirBD*. A detailed genomic analysis of MB3 will be carried out in the future to provide deeper insights into its specific biological function and ecological associations with mangroves.

### Accession number(s).

This whole-genome shotgun project has been deposited in DDBJ/ENA/GenBank under the accession no. MPIO00000000. The version described in this paper is the first version, MPIO01000000.
